# Elevated fatty acid amide hydrolase in the prefrontal cortex of borderline personality disorder: a [^11^C]CURB positron emission tomography study

**DOI:** 10.1038/s41386-020-0731-y

**Published:** 2020-06-10

**Authors:** N. J. Kolla, R. Mizrahi, K. Karas, C. Wang, R. M. Bagby, S. McMain, A. I. Simpson, P. M. Rusjan, R. Tyndale, S. Houle, I. Boileau

**Affiliations:** 1 Centre for Addiction and Mental Health (CAMH), Toronto, ON Canada; 2 Violence Prevention Neurobiological Research Unit, CAMH, Toronto, ON Canada; 3 Department of Psychiatry, University of Toronto, Toronto, ON Canada; 4 Department of Pharmacology and Toxicology, University of Toronto, Toronto, ON Canada; 5 Waypoint Centre for Mental Health Care, Penetanguishene, ON Canada

**Keywords:** Neuroscience, Translational research

## Abstract

Amygdala-prefrontal cortex (PFC) functional impairments have been linked to emotion dysregulation and aggression in borderline personality disorder (BPD). Fatty acid amide hydrolase (FAAH), the major catabolic enzyme for the endocannabinoid anandamide, has been proposed as a key regulator of the amygdala-PFC circuit that subserves emotion regulation. We tested the hypothesis that FAAH levels measured with [^11^C]CURB positron emission tomography in amygdala and PFC would be elevated in BPD and would relate to hostility and aggression. Twenty BPD patients and 20 healthy controls underwent FAAH genotyping (rs324420) and scanning with [^11^C]CURB. BPD patients were medication-free and were not experiencing a current major depressive episode. Regional differences in [^11^C]CURB binding were assessed using multivariate analysis of covariance with PFC and amygdala [^11^C]CURB binding as dependent variables, diagnosis as a fixed factor, and sex and genotype as covariates. [^11^C]CURB binding was marginally elevated across the PFC and amygdala in BPD (*p* = 0.08). In *a priori* selected PFC, but not amygdala, [^11^C]CURB binding was significantly higher in BPD (11.0%, *p* = 0.035 versus 10.6%, *p* = 0.29). PFC and amygdala [^11^C]CURB binding was positively correlated with measures of hostility in BPD (*r* > 0.4; *p* < 0.04). This study is the first to provide preliminary evidence of elevated PFC FAAH binding in any psychiatric condition. Findings are consistent with the model that lower endocannabinoid tone could perturb PFC circuitry that regulates emotion and aggression. Replication of these findings could encourage testing of FAAH inhibitors as innovative treatments for BPD.

## Introduction

Exploring novel neurotransmitter systems to better understand the pathology of disease states holds high translational potential. The discovery of the central endocannabinoid system (ECS), including cannabinoid receptors, their endocannabinoid (EC) lipid ligands, and enzymes regulating EC function, triggered a cascade of research investigating the relationship between abnormalities of the ECS and psychiatric symptomatology [1–7]. One target of interest is fatty acid amide hydrolase (FAAH), an intracellular, membrane-bound enzyme located postsynaptic to the cannabinoid receptor type 1 (CB1) that—among others—metabolizes anandamide (AEA) [8, 9]. AEA is arguably the most relevant endogenous peroxisome proliferator-activated gamma ligand and transcription factor of the nuclear hormone receptor superfamily [10], and it also binds to the transient receptor potential vanilloid type 1 [11]. AEA is also an EC whose primary molecular target is CB1 [12]. Produced on-demand in postsynaptic membranes before engaging in retrograde feedback onto presynaptic CB1 [13], AEA causes inhibition of specific neurotransmitter release. Alteration of FAAH enzyme activity indirectly regulates CB1 function through its actions on AEA [14]. Consequently, pharmaceuticals that modulate FAAH brain levels have been vigorously pursued [15, 16], especially in light of the problematic psychiatric side effects linked to CB1 antagonists or agonists like Δ^9^-tetrahydrocannabinol (THC) [17].

Borderline personality disorder (BPD) is a common mental condition that afflicts 10% of psychiatric outpatients and 20% of psychiatric inpatients [18]. It presents with dysregulated affective states, often manifested by hostility and anger, which predispose to high physical aggression [19, 20]. Treatment costs of BPD and lost productivity place a substantial economic burden on society, making BPD one of the most expensive mental disorders [21]. Although the mainstay of treatment for BPD is psychotherapy, pharmacological interventions play an important role in managing the disorder, evidenced by the fact that 40% of BPD patients take three or more psychotropic medications concurrently, 20% receive four or more medications, and 10% require greater than five psychotropic medications [22]. However, it is important to note that there are no FDA-approved medications for the treatment of BPD; further, most pharmaceuticals that are used off-label have not undergone rigorous testing. Reducing polypharmacy in BPD could be achieved by the development of new evidence-based pharmacotherapeutics with greater specificity for BPD pathophysiology and its symptom clusters.

In this regard, a leading hypothesis of the pathophysiology of BPD is that it is associated with abnormal function within the amygdala-PFC circuit, which regulates emotion control. Several functional neuroimaging studies of BPD with high hostility and poor emotion control have documented abnormal circuitry between regions of the PFC and the amygdala [23, 24]. In BPD, the PFC shows impairment in regulating higher-order decision-making and fails to withhold a response or revoke a planned action [25, 26] that then manifests as hypo-responsiveness in functional imaging studies. By contrast, heightened activity of the amygdala among patients with BPD may be related to the experience of more intense negative emotionality [27]. The PFC and amygdala are among the most common brain regions of interest (ROIs) showing abnormalities in BPD [28], hence the importance of prioritizing these regions in research paradigms. An emerging body of preclinical and neuroimaging studies suggests that abnormalities in EC signaling within this system may be involved in emotion dysregulation and aggression, which are core features of BPD [29, 30]. The current model proposes that exposure to stress or adversity increases FAAH-mediated AEA hydrolysis in amygdala-PFC circuitry, thereby leading to poor emotion regulation.

The data on the status of the ECS in BPD are inconsistent. For example, higher serum levels of AEA were detected in BPD patients, some of whom had comorbid posttraumatic stress disorder (PTSD). This group was compared with subjects who had PTSD alone as well as a cohort of healthy controls [4]. However, as members from each group were using cannabis and some patients were receiving psychotropic medications, these findings remain difficult to interpret. Moreover, at present, the relationship between plasma ECs and brain function remains poorly understood [31]. For example, in healthy participants, there is no relationship between peripheral plasma levels of AEA and cerebrospinal fluid (CSF) AEA levels [32]. On the other hand, AEA concentration in the hair was found to be lower in BPD [33]. Other data have signaled that AEA may be lower in the CSF of BPD [34], which is consistent with the notion that central levels of FAAH could be higher in BPD.

Based on the current model that increased FAAH is associated with dysfunction in the amygdala-PFC circuit believed to be impaired in BPD coupled with findings of lower AEA in BPD [33, 34], we hypothesized that FAAH levels, quantified using [^11^C]CURB positron emission tomography (PET), would be greater in the PFC and amygdala of BPD compared with healthy controls. We also conducted exploratory analyses to determine whether measures of hostility would show an association with FAAH levels in these same regions.

## Methods

All participants provided written informed consent after all study components were fully explained to them. All procedures were approved by the Research Ethics Board of the Centre for Addiction and Mental Health (CAMH) in Toronto, Ontario, Canada.

### Participants

Twenty patients with a diagnosis of BPD and 20 healthy controls recruited from the community completed the investigation. BPD participants were recruited from the local Toronto community and the outpatient Borderline Personality Disorder Clinic at CAMH. All experimental subjects had been previously diagnosed with BPD. Nevertheless, the diagnosis was verified according to results from the Structured Clinical Interview for DSM-IV Axis II Disorders [35] by trained raters. All diagnoses were reviewed and confirmed by a psychiatrist experienced in the assessment and treatment of personality disorders (NJK). Exclusion criteria for the BPD participants included a current major depressive episode (MDE); history of mania, hypomania, or psychotic illness; and diagnosis of substance abuse or dependence in the past 12 months as confirmed by the Structured Clinical Interview for DSM-IV Axis I Disorders [36]. We excluded individuals with a current MDE, as is the convention for many PET studies of BPD [37, 38], given the overlap in symptomatology between the two disorders and our desire to avoid this confound. Healthy controls were excluded if they had any history of psychiatric disorder. We also excluded cigarette smokers in both groups. In addition to self-report, nonsmoking status in BPD and in the healthy control participants was confirmed with a carbon monoxide breathalyzer (piCO Smokerlyzer, Bedford Scientific Ltd., Maidstone, UK), where subjects had consistent readings of <10 parts per million. The use of psychotropic medications or herbs in the past three months was also exclusionary for BPD and healthy participants. Neurological illness, head trauma, positive drug screen for drugs of abuse on scan and assessment days, pregnancy in females, and contraindications to safe magnetic resonance imaging (MRI) scanning also precluded participation. Similar to the experimental group, healthy controls were asked to refrain from using alcohol the night before and the day of PET scanning and were also asked not to drink caffeinated beverages on the day of the PET scan.

### Image acquisition and analysis

Each participant underwent one [^11^C]CURB PET scan at the CAMH Research Imaging Centre. [^11^C]CURB radiosynthesis has been previously described [39]. PET was completed with a three-dimensional HRRT brain tomograph (CPS/Siemens, Knoxville, TN, USA). Briefly, participants lay down on the scanning table with their heads immobilized with a thermoplastic mask to reduce movement. Next, a brief transmission scan was performed followed by injection of 370 ± 40 MBq (10 ± 1 mCi) of [^11^C]CURB [40]. [^11^C]CURB has been particularly noted for its high degree of specific binding following blocking with cold compounds and structurally-related compounds [41]. Brain radioactivity was quantified during sequential frames of increasing duration. The scan was 60 min. PET images were then reconstructed utilizing a filtered back-projection algorithm, with a HANN filter at Nyquist cutoff frequency [42]. After [^11^C]CURB injection, arterial samples were manually sampled from a radial artery at 3, 7, 12, 20, 30, 45, and 60 min and automatically for the first 22.3 min (automatic blood sampling system, Model PBS-101, Veenstra Instruments, The Netherlands). Manual and automatic arterial blood sampling were performed to assess the ratio of radioactivity in whole blood to radioactivity in plasma required to create the input function for the kinetic analysis [39]. Blood-to-plasma radioactivity ratios were interpolated by a biexponential function and parent plasma fraction by a Hill function.

Each participant completed a standard proton-density weighted brain MRI scan (TE = 17, TR = 6000, FOV = 22 cm, matrix = 256 × 256, slice thickness = 2 mm; number of excitations = 2) acquired on a Discovery MR750 3 T MRI scanner (General Electric, Milwaukee, WI, USA) for the purpose of ROI delineation. ROIs were automatically generated using the in-house software (ROMI) as previously described [43]. Time-activity curves were acquired over 60 min. in each ROI and analyzed by a two-tissue compartment model with irreversible binding to the second component [39]. The parameter of interest to quantify FAAH binding is the composite parameter λ*k*_3_ (λ = *K*_1_/*k*_2_).

### FAAH polymorphism genotyping

The binding affinity of [^11^C]CURB to FAAH is known to be affected by a single-nucleotide polymorphism in the human *FAAH* gene (rs324420) that involves a transversion of a cytosine to an adenine nucleoside (C385A) [44]. Relative to the C/C genotype, those homozygous or heterozygous for the A allele have reduced [^11^C]CURB binding (λ*k*_3_) in the brain [40]. For the BPD participants and healthy controls, the *FAAH* rs324420 variant was genotyped according to the manufacturer’s directions for a TaqMan SNP Genotyping assay (ID C_1897306_10; Life Technologies, Burlington, ON, Canada) on a ViiA7 instrument (Life Technologies, Burlington, ON, Canada) using 20 ng total genomic DNA template, Perfecta FastMix II (Quantabio, Beverly, MA, USA), in a total reaction volume of 10 uL as previously performed [44].

### Instruments

BPD subjects completed the Buss-Durkee Hostility Inventory (BDHI) [45] to assess trait aggressiveness. The Beck Depression Inventory [46] and State Trait Anxiety Inventory measured depressive and anxiety symptoms, respectively [47]. BPD subjects were also administered the Zanarini Rating Scale for Borderline Personality Disorder (ZAN-BPD) to assess severity of BPD [48]. In addition, BPD participants completed the Childhood Trauma Questionnaire-Short Form (CTQ-SF) [49] that retrospectively identifies subjects’ experience of emotional neglect, emotional abuse, physical neglect, physical abuse, and sexual abuse during childhood.

### Neuropsychology tests

The Iowa Gambling Task (IGT) is a performance-based card game that indexes choice impulsivity [50]. It has been used extensively in BPD as a means to assess disadvantageous decision-making and PFC function [51, 52]. Successful performance relies on intact orbitofrontal cortex function [53]. Participants were encouraged to win as much virtual money as possible by selecting cards from four decks (A, B, C, or D) one at a time. Decks C and D produce high monetary gains and risk of high losses, whereas decks A and B yield lower gains with the risk of smaller losses. Research subjects were told that some decks are less advantageous than others and that they could win by avoiding these decks. Twenty trials were administered over five blocks for a total of 100 trials. Highly impulsive groups show the greatest impairment in performance during the latter trials of the IGT [54]. Accordingly, the net IGT was calculated by subtracting the number of cards selected from disadvantageous decks from the number of cards selected from advantageous decks over the last two blocks: ([C + D]-[A + B]) [55]. We compared PFC [^11^C]CURB λ*k*_3_ with these two IGT outcome measures.

### Statistical analysis

Sample demographic information was compared between groups using chi-square tests and independent samples *t* tests. To compare [^11^C]CURB λ*k*_3_ between groups (BPD versus healthy controls), we employed full factorial multivariate analysis of covariance (MANCOVA) with diagnosis as a fixed factor and genotype as a covariate. Because the sex distribution differed between groups (see below), we additionally included sex as a covariate for the multivariate analyses. However, we also conducted analyses without sex as a covariate. The main model that was used to test our primary hypothesis included two ROIs: PFC and amygdala. Main effects were analyzed by univariate ANOVA with a *p* value of less than 0.05 indicating significance. Our secondary model tested the whole brain including anterior cingulate cortex (ACC), temporal cortex, insula, hippocampus, ventral striatum, dorsal putamen, dorsal caudate, and cerebellum (10 ROIs). Bonferroni correction was applied (0.05/8 secondary regions = 0.0063) to correct for multiple comparisons.

Partial correlations controlling for FAAH genotype were conducted as exploratory analyses to quantify the relationship between [^11^C]CURB λ*k*_3_ in the hypothesized ROIs and hostility.

## Results

### Subject characteristics

Participants’ clinical and demographic information is reported in Table 1 (Information on comorbid conditions is presented in Supplementary Table 1). Participants were aged 18–47 years. A subset of healthy participants (*n* = 8) had previously participated in other PET experiments [2]. There were more females in the BPD group (*n* = 18) compared with the healthy control group (*n* = 10). Groups did not differ in terms of age, ethnicity, years of education, or body mass index. Ninety percent of BPD participants had major depressive disorder (no current MDE), while 20% had current PTSD. BPD participants were matched to healthy controls on genotype for the *FAAH* gene polymorphism (rs324420; C/C carriers versus A/C or A/A carriers).

**Table 1:** Clinical and demographic variables.

	BPD (*n* = 20)	Controls (*n* = 20)	Statistics	*p* value
Age (years)^a^	28.0 ± 8.4	25.7 ± 8.3	*t* = 0.87	0.39
Sex (F/M)^b^	18/2	10/10	χ^2^ = 7.6	0.006
Ethnicity^b^	/	/	χ^2^ = 3.7	0.45
Caucasian (#)	12	7	/	/
Black (#)	2	5	/	/
Middle Eastern (#)	0	1	/	/
South Asian (#)	2	2	/	/
Asian (#)	4	5	/	/
Education (years)^a^	14.5 ± 2.4	15.3 ± 1.8	*t* = −1.2	0.24
Genotype (CC or CA/AA)^b^	17/3	17/3	χ^2^ = 0	1.0
Body mass index^a^	25.3 ± 6.0	23.4 ± 3.9	*t* = 1.2	0.24
Self-Harm				
% Cutting self (in past year)	75%	/	/	/
% Burning self (in past year)	35%	/	/	/
% Banging/hitting self (in past year)	85%	/	/	/
% Overdose (in past year)	45%	/	/	/
BDHI^c^				
Assault	5.0 ± 3.2	/	/	/
Indirect	6.0 ± 2.5	/	/	/
Irritability	8.6 ± 2.2	/	/	/
Negativity	3.3 ± 1.5	/	/	/
Resentment	5.6 ± 1.5	/	/	/
Suspicion	5.2 ± 2.5	/	/	/
Verbal	8.9 ± 3.2	/	/	/
Guilt	5.3 ± 2.1	/	/	/
Total	42.4 ± 12.0	/	/	/
ZAN-BPD^d^	15.3 ± 4.7	/	/	/
BDI^e^	21.7 ± 12.4	/	/	/
STAI^f^				
Trait	64.6 ± 5.7	/	/	/
State	48.7 ± 11.1	/	/	/
CTQ-SF^g^				
Emotional neglect	3.6 ± 0.8	/	/	/
Emotional abuse	3.6 ± 1.1	/	/	/
Physical neglect	2.2 ± 0.8	/	/	/
Physical abuse	2.2 ± 1.2	/	/	/
Sexual abuse	1.8 ± 1.4	/	/	/
Tracer specific activity (mCi/μmol)^a^	4149.7 ± 1110	4174.7 ± 2348	*t* = −0.043	0.96

^a^Independent samples *t* test.

^b^chi-square test.

^c^Buss-Durkee Hostility Inventory.

^d^Zanarini Rating Scale for Borderline Personality Disorder.

^e^Beck Depression Inventory.

^f^State Trait Anxiety Inventory.

^g^Childhood Trauma Questionnaire-Short Form.

### Comparison of [^11^C]CURB λ*k*_3_ in BPD and healthy control groups

We observed a trend toward an overall effect of diagnosis on [^11^C]CURB λ*k*_3_ across the two hypothesized ROIs (*F*_2,32_ = 2.7, *p* = 0.08). When we reran the analyses without sex as a covariate, results remained unchanged (*F*_2,32_ = 2.2, *p* = 0.13). Using the effect of diagnosis in the ANCOVA for our two hypothesized ROIs (PFC and amygdala), results revealed that BPD participants had 11.0% greater PFC [^11^C]CURB λ*k*_3_ compared with healthy controls (0.16 ± 0.025 versus 0.14 ± 0.024, *F*_1,33_ = 4.8, *p* = 0.035, *η*^2^ = 0.11). Results remained significant with the exclusion of sex as a covariate (*F*_1,36_ = 4.3, *p* = 0.044). There was no significant difference in amygdala [^11^C]CURB λ*k*_3_ between groups (10.6% elevation; 0.15 ± 0.020 versus. 0.14 ± 0.029, *F*_1,33_ = 1.8, *p* = 0.29). Results similarly did not reach significance when sex was removed as a covariate (*F*_1,36_ = 3.2, *p* = 0.083).

**Fig. 1:**
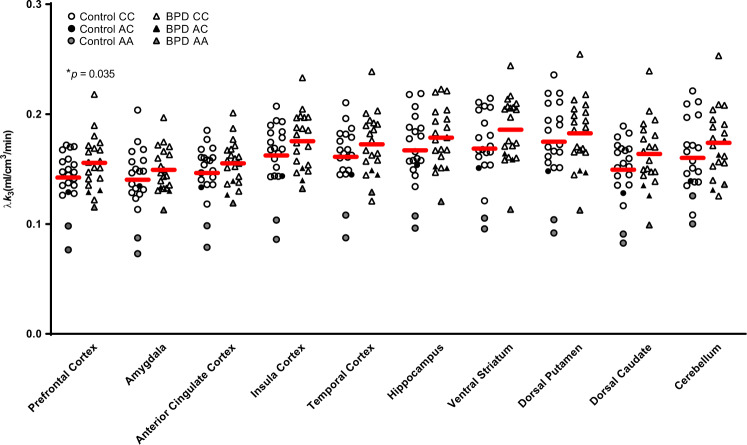
Elevation of prefrontal cortex fatty acid amide hydrolase λ*k*_3_ in borderline personality disorder. After controlling for genotype, borderline personality disorder subjects were found to have greater fatty acid amide hydrolase density in the prefrontal cortex compared with healthy controls.

No significant effect of group was detected when we sampled the 10 ROIs for the whole brain analysis (*F*_10,24_ = 1.6, *p* = 0.16; percentage of elevated FAAH binding in BPD for each ROI: 10.6–11.2%). When sex was removed as a covariate, results were the same (*F*_10,27_ = 1.1, *p* = 0.40). Many of the secondary ROIs had *p* values < 0.05; however, none survived Bonferroni correction. These results are presented graphically in Fig. 1 (Statistics for secondary ROIs are presented in Table 2). As expected, there was a main effect of genotype for both sets of analyses (*p* < 0.01). There were no significant interactions.

**Table 2:** Statistics for secondary regions of interest.

Secondary regions of interest (BPD versus Controls)	Type III sum of squares	*F*	Degrees of freedom	*p* value
Anterior cingulate cortex	0.002	5.0	1,33	0.032
Insula cortex	0.001	5.1	1,33	0.031
Temporal cortex	0.003	5.0	1,33	0.032
Hippocampus	0.002	3.4	1,33	0.075
Dorsal caudate	0.003	5.4	1,33	0.026
Dorsal putamen	0.002	3.8	1,33	0.059
Ventral striatum	0.004	6.7	1,33	0.014
Cerebellum	0.001	1.4	1,33	0.25

### Effect of PTSD and anxiety on relationship between PFC and amygdala [^11^C]CURB λ*k*_3_

Given evidence implicating FAAH in the pathogenesis of PTSD [56] and the high rate of comorbidity between PTSD and BPD, we used MANCOVA to test the effect of comorbid PTSD on PFC and amygdala [^11^C]CURB λ*k*_3_. Twenty percent of the BPD sample screened positive for current PTSD (Supplementary Table 1). There was no significant main effect of PTSD (*F*_2,31_ = 0.49, *p* = 0.62). Furthermore, the effect of BPD diagnosis remained significant for PFC FAAH λ*k*_3_ (*F*_1,32_ = 5.0, *p* = 0.032), but not amygdala FAAH λ*k*_3_ (*F*_1,32_ = 1.3, *p* = 0.27), after controlling for genotype, sex, and the presence of PTSD.

We similarly tested whether [^11^C]CURB λ*k*_3_ differed between each of the 10 ROIs for the following contrasts: (1) BPD with PTSD versus BPD without PTSD; (2) BPD with generalized anxiety disorder (GAD) versus BPD without GAD; (3) BPD with social anxiety disorder (SAD) versus BPD without SAD; and (4) BPD with specific phobia (SP) versus BPD without SP. There were no significant differences for any of the 10 ROIs (all *p* values > 0.05).

### Partial correlations between [^11^C]CURB λ*k*_3_ and hostility, depression, state and trait anxiety, CTQ-SF scores, and IGT measures

**Fig. 2:**
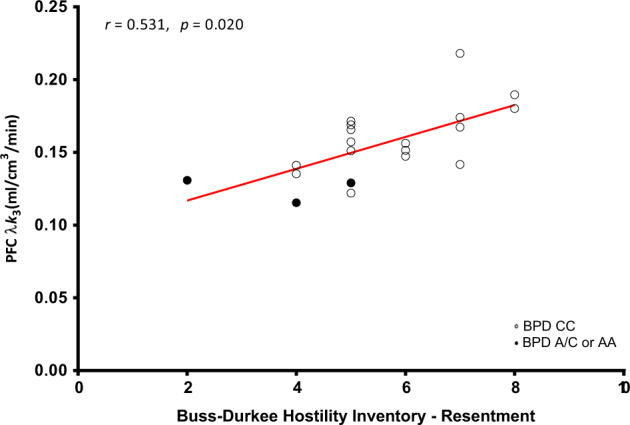
Prefrontal cortex fatty acid amide hydrolase λ*k*3 is correlated with hostility (Resentment) controlling for genotype in borderline personality disorder. That is, borderline personality disorder subjects who had higher scores on the Resentment subscale of the Buss-Durkee Hostility Inventory also had higher fatty acid amide hydrolase expression in the prefrontal cortex.

We first tested the partial correlations between PFC and amygdala [^11^C]CURB λ*k*_3_ and BDHI subscales and total score controlling for genotype. We found a significant correlation between PFC [^11^C]CURB λ*k*_3_ and the Resentment subscale score of the BDHI (*r* = 0.53, *p* = 0.020; Fig. 2). None of the other correlations of PFC with BDHI subscales or total scores were significant (all *p* values > 0.05).

**Fig. 3:**
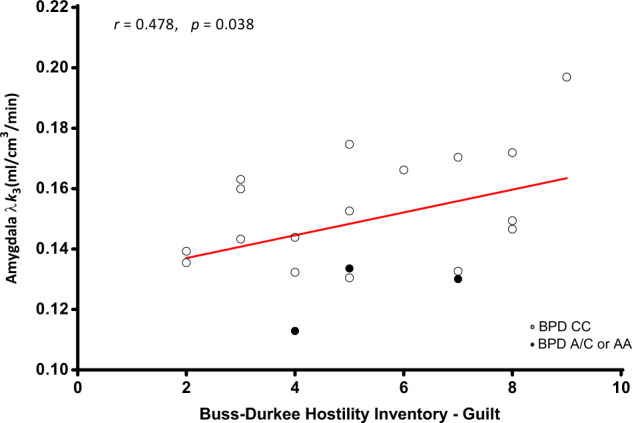
Amygdala fatty acid amide hydrolase λ*k*3 is correlated with hostility (Guilt) controlling for genotype in borderline personality disorder. Borderline personality disorder participants who scored higher on Guilt subscale scores of the Buss-Durkee Hostility Inventory had greater fatty acid amide hydrolase density in the amygdala.

We similarly discerned a significant correlation between amygdala [^11^C]CURB λ*k*_3_ and the BDHI Guilt subscale score (*r* = 0.48, *p* = 0.038; Fig. 3).

None of the other correlations of amygdala with BDHI subscale scores were significant (all *p* values > 0.05). In addition, ACC [^11^C]CURB λ*k*_3_ (*r* = 0.52, *p* = 0.021), insula [^11^C]CURB λ*k*_3_ (*r* = 0.48, *p* = 0.039), and dorsal caudate [^11^C]CURB λ*k*_3_ (*r* = 0.47, *p* = 0.045) were all significantly correlated with the Resentment subscale score of the BDHI.

None of the 10 ROI [^11^C]CURB λ*k*_3_ values were significantly correlated with ZAN-BPD, CTQ-SF, depression, or anxiety scores after applying Bonferroni correction. Furthermore, PFC FAAH binding did not correlate with IGT decks ([C + D]-[A + B]) over the second last (*r* = 0.15, *p* = 0.56) and last blocks (*r* = 0.21, *p* = 0.43).

## Discussion

To the best of our knowledge, this study is the first to document an elevation of PFC FAAH ([^11^C]CURB λ*k*_3_) assessed using PET in any psychiatric disorder. Specifically, we found that BPD participants who were medication-free, nonsubstance using, nonsmoking, had no alcohol use disorder, were not experiencing an MDE, but were still engaging in non-suicidal self injury, had higher FAAH levels in the PFC compared with healthy controls. Elevated PFC FAAH persisted in BPD even after controlling for comorbid PTSD and anxiety disorders. BPD subjects also showed positive correlations between PFC, ACC, insula, dorsal caudate, and amygdala FAAH binding with measures of anger/hostility. As far as we are aware, the present study is the only PET investigation of BPD to assay a neurochemical target beyond the opioid and monoaminergic systems. Nonetheless, these results must be tempered by the relatively small sample size.

The increase in [^11^C]CURB λ*k*_3_ was not restricted or localized to the PFC. Figure 1 generally reveals the same order of magnitude increase (10.6–11.2%) across the board in all 10 ROIs that were examined, where regional estimates of ROIs were also similar. Similarly, a global elevation of brain CB1 availability, including the amygdala and orbitofrontal cortex, has been reported in PTSD, a trauma and stressor-related disorder that shares some clinical similarities with BPD [57]. In general, regional levels of brain FAAH tend to be correlated [39] and specific binding is very high for [^11^C]CURB [41]. We suggest that a global binding increase of [^11^C]CURB λ*k*_3_ may be operating in BPD with self-harming behavior. However, these results need to be replicated by independent groups to enhance these conclusions. There are very few FAAH PET studies that have sampled psychiatric patients. Yet, studies of chronic cannabis users and individuals with alcohol use disorders revealed a global decrease in [^11^C]CURB λ*k*_3_ among all ROIs tested [1, 2]. It may be that brain markers of FAAH show differential but correlated responses in certain psychopathologies. Quantifying [^11^C]CURB λ*k*_3_ in patients with other psychiatric disorders would help test this hypothesis.

2-Arachidonoylglycerol (2-AG) is an endogenous lipid ligand that may also be implicated in the pathogenesis of BPD given that 2-AG signaling affects stress adaptation and anxiety- or depressive-like behaviors in rodent models [58]. 2-AG is predominantly hydrolyzed by monoacylglycerol lipase (MAGL) [59]. Since MAGL inhibition has been shown to reduce anxiety- and depressive-like behaviors in preclinical models [60], therapeutics that employ MAGL inhibition could also emerge as novel treatments for BPD.

Several investigations have proposed that failure of prefrontal regulatory control mechanisms to modulate limbic hyperreactivity in BPD may predispose to hostility, impulsive aggression, and aberrant emotional processing [23, 24]. These symptoms may also occur as a result of dysfunctional prefrontal serotonergic regulation that promotes amygdala-driven emotional behavior [61]. Indeed, treatment of BPD with selective serotonin (5-HT) reuptake inhibitors increases metabolic activity in the PFC and also attenuates impulsive aggression [62]. Preclinical studies have demonstrated that inhibition or genetic deletion of FAAH increases AEA, which in turn binds to CB1 to facilitate serotonergic neurotransmission, ultimately leading to antidepressant and anxiolytic effects [63, 64]. Since FAAH inhibition is associated with increased AEA and amplification of serotonergic tone [65], we suggest that elevated FAAH, in contrast, may be a cause of reduced AEA, leading to decreased CB1 signaling, dampened serotonergic transmission, and enhanced expression of BPD symptomatology. Interestingly, preclinical designs involving sub-chronic or chronic exposure of Δ^9^-THC produce elevated 5-HT_1A_ receptor activity [66] and upregulation of 5-HT_2A_ receptors in rat PFC [67], providing further evidence of the primacy of CB1 activation in moderating serotonergic neurotransmission. Critically, there are currently no pharmaceuticals that are FDA-approved for the treatment of BPD. We propose that our results provide preliminary evidence for the testing of highly selective and reversible FAAH inhibitors as potential new pharmacological treatments for BPD.

We also found that PFC and amygdala [^11^C]CURB λ*k*_3_ in addition to other ROIs were positively correlated with measures of anger/hostility. These analyses were exploratory and would not survive Bonferroni correction. However, given that an EC signaling system may be relevant to manifestation of aggression [68], we believe that it is justified to pursue these results. Hostility in BPD is also highly clinically relevant, as its expression is linked to the presence, number, and severity of suicide attempts [69], in addition to completed suicide in this population [70]. Δ^9^-THC has been shown to reduce hostile feelings within small social group settings [71], suggesting a role for the ECS and potentially other signaling pathways in mediating anger/hostility. Recent research has focused on dysregulated oxytocin function in BPD as one possible mechanism of anger/hostility. Oxytocin is a neuropeptide that has been shown to increase social affiliation and prosocial behaviors in both animals and humans [72]. Meta-analysis indicates that oxytocin also increases functional connectivity between the PFC and amygdala [73]. In BPD, oxytocin may attenuate hypervigilance to interpersonal threat and decrease amygdala hyperactivity [74]. Interestingly, preclinical data report connections between oxytocin and ECS function. Oxytocin promotes AEA signaling at CB1 in the nucleus accumbens to influence the rewarding properties of social connections. By interrupting AEA degradation, FAAH counterbalances the effects of oxytocin receptor blockade on social place preference and nucleus accumbens cFos expression [75]. For instance, administration of an oxytocin antagonist to wild-type mice reduced social conditioned place preference, whereas mice with genetic deletion of FAAH were unaffected by oxytocin antagonism and engaged in the rewarding properties of social behavior [75]. These results suggest that lower FAAH brain levels may be critical for engaging in social reward and suppression of anger/hostility. We propose that the anger and hostility characteristic of BPD could reflect the interaction of increased FAAH with deficient oxytocin signaling. Further investigations, including measurement of central oxytocin levels, would be pivotal to test this hypothesis.

We found no relationship between comorbid anxiety disorders, depressive symptoms, or state or trait measures of anxiety and [^11^C]CURB λ*k*_3_ for any of the ROIs tested, although admittedly the sample is relatively small. These comorbidities may not explain our findings, but we do not rule out the possibility that FAAH may be dysregulated in anxiety disorders. Nevertheless, the observed correlations between PFC and amygdala [^11^C]CURB λ*k*_3_ with measures of hostility suggest that this symptom of BPD may be uniquely related to corticolimbic FAAH levels.

We note several limitations of this investigation. First, this design cannot inform on whether the observed elevation of PFC [^11^C]CURB λ*k*_3_ in BPD is a state or trait marker of psychopathology. Future research should endeavor to assay PFC [^11^C]CURB λ*k*_3_ longitudinally to discern whether this biomarker is sensitive to change following clinical improvement (e.g., cessation of self-harming behavior). Second, we excluded BPD patients with certain comorbidities and those who were taking medication to eliminate potential confounds. Although we excluded patients who suffered from polymorphic psychotic symptoms and psychotic illness, the majority of the BPD participants met diagnostic criteria for dissociative experiences and transient paranoid ideation (e.g., intermittent psychotic symptoms). For example, we additionally tested whether BPD participants who endorsed micropsychotic symptoms (e.g., transient, stress-related paranoid ideation or severe dissociative symptoms—one DSM-5 criterion for BPD) had differences in FAAH binding for each ROI compared with those who did not experience micropsychotic symptoms. Seventeen BPD participants endorsed micropsychotic phenomena, whereas the remaining three individuals did not have micropsychotic symptoms. There was no difference in [^11^C]CURB λ*k*_3_ between groups (data not shown). Thus, it could be argued that most of the subjects we sampled had mild forms of BPD, given that participants experienced micropsychotic symptoms but had no formal psychotic disorder. However, all subjects were actively self-harming, and the mean ZAN-BPD score was 15.3, which is similar to scores of BPD patients engaging in pharmacological treatment studies (17.0) [76], suggesting that BPD severity in our sample approximated the acuity of the larger population of BPD participants requiring pharmacological treatment. We also did not collect data on serum levels of ECs, such as AEA, and the N-acylethanolamides N-palmitoylethanolamine and N-oleoylethanolamine that may have shown some correlation with FAAH activity in vivo. However, brain concentrations of ECs do not correlate with peripheral concentrations in a clear-cut manner due to excess physiological factors, which currently hinders the usefulness of inferring brain activity from peripheral measures [31]. Another limitation is that we did not include clinician-administered instruments in our testing battery designed to assess for depressive symptoms. Ideally, both self-report and clinician-administered instruments should be used to assess depression severity [77]. Finally, we were unable to completely match participants on the lower affinity C385A polymorphism; that is, the control group had two A/A subjects, whereas only one BPD subject had the A/A polymorphism. However, controlling for genotype did not affect the finding. We were unable to match on sex, because we required healthy controls who met very strict inclusion and exclusion criteria (e.g., no axis I or II disorder, nonsmoking, no drug use). Therefore, we treated sex as a covariate in the analyses. There are also published [^11^C]CURB PET data from our laboratory that show no effect of sex on [^11^C]CURB λ*k*_3_ in healthy and psychiatric populations (*n* = 66; 32 males and 34 females matched for age and body mass index; *F*_1,58_ = 0.57, *p* = 0.45) [1]. Finally, Of the 18 female BPD participants, nine were in the luteal phase of their menstrual cycle, six were in the follicular phase, and three suffered from amenorrhea (one suffered from polycystic ovarian syndrome, another was taking an injectable birth control medication, and the third had recently given birth). No relationship was detected between phase of menstrual cycle and FAAH binding (data not shown). Sex hormones would also be informative to test.

In conclusion, PET studies of BPD are sparse in the literature, perhaps due to the necessity of enforcing stringent inclusion and exclusion criteria [23]. Yet, the information gleaned from PET investigations is vital for characterizing novel neurochemical targets amenable to pharmacological intervention. Here, we report for the first time a small elevation of brain FAAH binding in BPD, a condition with few identified neurochemical biomarkers to date. Specifically, we found that PFC FAAH binding was elevated by 11% in BPD and that PFC and amygdala FAAH binding were positively correlated with hostility/anger, although results must be interpreted with caution given the smaller sample size. Subject to replication, these results might encourage investigation of reversible FAAH inhibitors as potential new treatment alternatives for BPD and stimulate research investigating FAAH status in conditions often co-occurring with BPD, such as major depressive disorder and substance use disorders, for which therapeutic modulation of FAAH has been proposed and the evidence base is currently stronger.

## Funding and disclosure

This work was funded by a Canadian Institutes of Health Research (CIHR) Operating Grant and a CIHR Clinician Scientist Salary Award, both awarded to NJK; the Canada Research Chair Program and a CIHR Foundation Grant (FDN: 154294), awarded to RT; funding from the Centre for Addiction and Mental Health (CAMH) and CAMH Foundation, awarded to NJK and RT; and an Ontario Mental Health Foundation Operating Grant and a NIDA R21 Grant (DA036024-01) awarded to IB. All authors report no disclosures except for RT who has consulted for Quinn Emanuel and Ethismos Research Inc. during the past three years. None of these organizations had any input regarding the design and administration of this study or the interpretation of its results.

## Supplementary information

Supplementary Material

## References

[1] Best LM, Williams B, Le Foll B, Mansouri E, Bazinet RP, Lin L, et al. (2020). Lower brain fatty acid amide hydrolase in treatment-seeking patients with alcohol use disorder: a positron emission tomography study with [C-11]CURB. Neuropsychopharmacology. 2020:1–8. 10.1038/s41386-020-0606-2 PMC7298050 31910433

[2] Boileau I, Mansouri E, Williams B, Le Foll B, Rusjan P, Mizrahi R (2016). Fatty acid amide hydrolase binding in brain of cannabis users: imaging with the novel radiotracer [(11)C]CURB. Biol Psychiatry.

[3] Borgan F, Laurikainen H, Veronese M, Marques TR, Haaparanta-Solin M, Solin O, et al. (2019). In vivo availability of cannabinoid 1 receptor levels in patients with first-episode psychosis. JAMA Psychiatry. 2020;76:1074–84. 10.1001/jamapsychiatry.2019.1427 PMC6613300 31268519

[4] Schaefer C, Enning F, Mueller JK, Bumb JM, Rohleder C, Odorfer TM (2014). Fatty acid ethanolamide levels are altered in borderline personality and complex posttraumatic stress disorders. Eur Arch psychiatry Clin Neurosci.

[5] Wong DF, Kuwabara H, Horti AG, Raymont V, Brasic J, Guevara M (2010). Quantification of cerebral cannabinoid receptors subtype 1 (CB1) in healthy subjects and schizophrenia by the novel PET radioligand [11C]OMAR. NeuroImage.

[6] Bedse G, Bluett RJ, Patrick TA, Romness NK, Gaulden AD, Kingsley PJ (2018). Therapeutic endocannabinoid augmentation for mood and anxiety disorders: comparative profiling of FAAH, MAGL and dual inhibitors. Transl Psychiatry.

[7] Berardi A, Schelling G, Campolongo P (2016). The endocannabinoid system and Post Traumatic Stress Disorder (PTSD): From preclinical findings to innovative therapeutic approaches in clinical settings.. Pharmacol Res.

[8] Deutsch DG, Chin SA (1993). Enzymatic synthesis and degradation of anandamide, a cannabinoid receptor agonist. Biochemical Pharmacol.

[9] Schmid PC, Zuzarte-Augustin ML, Schmid HH (1985). Properties of rat-liver N-acylethanolamine amidohydrolase. J Biol Chem.

[10] Bouaboula M, Hilairet S, Marchand J, Fajas L, Le Fur G, Casellas P (2005). Anandamide induced PPAR gamma transcriptional activation and 3T3-L1 preadipocyte differentiation. Eur J Pharmacol.

[11] Tóth A, Blumberg PM, Boczán J (2009). Anandamide and the vanilloid receptor (TRPV1). Vitam Horm.

[12] Pertwee RG (2008). Ligands that target cannabinoid receptors in the brain: from THC to anandamide and beyond. Addiction Biol.

[13] Kano M, Ohno-Shosaku T, Hashimotodani Y, Uchigashima M, Watanabe M (2009). Endocannabinoid-mediated control of synaptic transmission. Physiological Rev.

[14] Vinod KY, Kassir SA, Hungund BL, Cooper TB, Mann JJ, Arango V (2010). Selective alterations of the CB1 receptors and the fatty acid amide hydrolase in the ventral striatum of alcoholics and suicides. J Psychiatr Res.

[15] Ahn K, Johnson DS, Cravatt BF (2009). Fatty acid amide hydrolase as a potential therapeutic target for the treatment of pain and CNS disorders. Expert Opin Drug Disco.

[16] Wang Y, Zhang X (2017). FAAH inhibition produces antidepressant-like efforts of mice to acute stress via synaptic long-term depression. Behavioural brain Res.

[17] Mitchell PB, Morris MJ (2007). Depression and anxiety with rimonabant. Lancet.

[18] Torgersen S, Kringlen E, Cramer V (2001). The prevalence of personality disorders in a community sample. Arch Gen Psychiatry.

[19] Kolla NJ, Meyer JH, Bagby RM, Brijmohan A (2017). Trait anger, physical aggression, and violent offending in antisocial and borderline personality disorders. J Forensic Sci.

[20] Leichsenring F, Leibing E, Kruse J, New AS, Leweke F (2011). Borderline personality disorder. Lancet.

[21] Wunsch EM, Kliem S, Kröger C (2014). Population-based cost-offset estimation for the treatment of borderline personality disorder: projected costs in a currently running, ideal health system. Behav Res Ther.

[22] Zanarini MC, Frankenburg FR, Hennen J, Silk KR (2004). Mental health service utilization by borderline personality disorder patients and Axis II comparison subjects followed prospectively for 6 years.. J Clin Psychiatry.

[23] New AS, Hazlett EA, Buchsbaum MS, Goodman M, Mitelman SA, Newmark R (2007). Amygdala-prefrontal disconnection in borderline personality disorder. Neuropsychopharmacology.

[24] Schmitt R, Winter D, Niedtfeld I, Herpertz SC, Schmahl C (2016). Effects of psychotherapy on neuronal correlates of reappraisal in female patients with borderline personality disorder. Biol Psychiatry Cogn Neurosci Neuroimaging.

[25] Arnsten AF (2009). Stress signalling pathways that impair prefrontal cortex structure and function. Nat Rev Neurosci.

[26] Widge AS, Heilbronner SR, Hayden BY. Prefrontal cortex and cognitive control: new insights from human electrophysiology. F1000Res. 2019;8. 10.12688/f1000research.20044.1 PMC6768099 31602292

[27] Ruocco AC, Amirthavasagam S, Choi-Kain LW, McMain SF (2013). Neural correlates of negative emotionality in borderline personality disorder: an activation-likelihood-estimation meta-analysis. Biol Psychiatry.

[28] Krause-Utz A, Winter D, Niedtfeld I, Schmahl C (2014). The latest neuroimaging findings in borderline personality disorder. Curr Psychiatry Rep..

[29] Hariri AR, Gorka A, Hyde LW, Kimak M, Halder I, Ducci F (2009). Divergent effects of genetic variation in endocannabinoid signaling on human threat- and reward-related brain function. Biol Psychiatry.

[30] Hill MN, McEwen BS (2010). Involvement of the endocannabinoid system in the neurobehavioural effects of stress and glucocorticoids. Prog Neuropsychopharmacol Biol Psychiatry.

[31] Hillard CJ (2018). Circulating endocannabinoids: from whence do they come and where are they going?. Neuropsychopharmacology.

[32] Giuffrida A, Leweke FM, Gerth CW, Schreiber D, Koethe D, Faulhaber J (2004). Cerebrospinal anandamide levels are elevated in acute schizophrenia and are inversely correlated with psychotic symptoms. Neuropsychopharmacology.

[33] Wingenfeld K, Dettenborn L, Kirschbaum C, Gao W, Otte C, Roepke S (2018). Reduced levels of the endocannabinoid arachidonylethanolamide (AEA) in hair in patients with borderline personality disorder—a pilot study. Stress.

[34] Koethe D, Schwarz E, Schaefer C, Enning F, Mueller JK, Bumbe JM, et al. Endocannabinoids and neuropeptides in CSF and serum from borderline personality disorder. Society of Biological Psychiatry. New York: New York; 2014.

[35] First MB, Gibbon M, Spitzer RL, Williams JBW, Benjamin LS. Structured Clinical Interview for DSM-IV Axis II Personality Disorders, (SCID-II) American Psychiatric Press, Inc.: Washington, DC; 1997.

[36] First MB, Spitzer RL, Gibbon M, Williams JBW. Structured Clinical Interview for DSM-IV-TR Axis I Disorders, Research Version, Patient Edition (SCID-I/P), Version 2. Biometrics Research, New York State Psychiatric Institute: New York, NY; 2002.

[37] Leyton M, Okazawa H, Diksic M, Paris J, Rosa P, Mzengeza S (2001). Brain Regional alpha-[11C]methyl-L-tryptophan trapping in impulsive subjects with borderline personality disorder.. Am J Psychiatry.

[38] Soloff PH, Price JC, Meltzer CC, Fabio A, Frank GK, Kaye WH (2007). 5HT2A receptor binding is increased in borderline personality disorder. Biol Psychiatry.

[39] Rusjan PM, Wilson AA, Mizrahi R, Boileau I, Chavez SE, Lobaugh NJ (2013). Mapping human brain fatty acid amide hydrolase activity with PET. J Cereb Blood Flow Metab.

[40] Boileau I, Bloomfield PM, Rusjan P, Mizrahi R, Mufti A, Vitcu I (2014). Whole-body radiation dosimetry of 11C-carbonyl-URB694: a PET tracer for fatty acid amide hydrolase. J Nucl Med.

[41] McCluskey SP, Plisson C, Rabiner EA, Howes O. Advances in CNS PET: the state-of-the-art for new imaging targets for pathophysiology and drug development. Eur J Nucl Med Mol Imaging. 2019;47:1–39. 10.1007/s00259-019-04488-0 PMC6974496 31541283

[42] Mizrahi R, Rusjan PM, Kennedy J, Pollock B, Mulsant B, Suridjan I (2012). Translocator protein (18 kDa) polymorphism (rs6971) explains in-vivo brain binding affinity of the PET radioligand [(18)F]-FEPPA. J Cereb Blood Flow Metab.

[43] Rusjan P, Mamo D, Ginovart N, Hussey D, Vitcu I, Yasuno F (2006). An automated method for the extraction of regional data from PET images. Psychiatry Res.

[44] Boileau I, Tyndale RF, Williams B, Mansouri E, Westwood DJ, Le Foll B (2015). The fatty acid amide hydrolase C385A variant affects brain binding of the positron emission tomography tracer [11C]CURB. J Cereb Blood Flow Metab.

[45] Buss AH, Durkee A (1957). An inventory for assessing different kinds of hostility. J Consulting Psychol.

[46] Beck AT, Ward CH, Mendelson M, Mock J, Erbaugh J (1961). An inventory for measuring depression. Arch Gen Psychiatry.

[47] Spielberger CD, Gorsuch, RL, Lushene, R, Vagg, PR, Jacobs, GA. Manual for the State-Trait Anxiety Inventory (form Y). Consulting Psychologists Press: Palo Alto, CA; 1983.

[48] Zanarini MC, Vujanovic AA, Parachini EA, Boulanger JL, Frankenburg FR, Hennen J (2003). Zanarini Rating Scale for Borderline Personality Disorder (ZAN-BPD): a continuous measure of DSM-IV borderline psychopathology. J Personal Disord.

[49] Bernstein DP, Stein JA, Newcomb MD, Walker E, Pogge D, Ahluvalia T (2003). Development and validation of a brief screening version of the childhood trauma questionnaire. Child Abus Negl.

[50] Bechara A, Damasio AR, Damasio H, Anderson SW (1994). Insensitivity to future consequences following damage to human prefrontal cortex. Cognition.

[51] Ouerchefani R, Ouerchefani N, Allain P, Ben Rejeb MR, Le Gall D (2019). Relationships between executive function, working memory, and decision-making on the Iowa Gambling Task: Evidence from ventromedial patients, dorsolateral patients, and normal subjects. J Neuropsychol.

[52] Paret C, Jennen-Steinmetz C, Schmahl C (2017). Disadvantageous decision-making in borderline personality disorder: Partial support from a meta-analytic review. Neurosci Biobehav Rev.

[53] Shurman B, Horan WP, Nuechterlein KH (2005). Schizophrenia patients demonstrate a distinctive pattern of decision-making impairment on the Iowa gambling task. Schizophrenia Res.

[54] Sweitzer MM, Allen PA, Kaut KP (2008). Relation of individual differences in impulsivity to nonclinical emotional decision making. J Int Neuropsychological Soc.

[55] Kolla NJ, Matthews B, Wilson AA, Houle S, Bagby RM, Links P (2015). Lower monoamine oxidase-A total distribution volume in impulsive and violent male offenders with antisocial personality disorder and high psychopathic traits: An [(11)C] harmine positron emission tomography study. Neuropsychopharmacol.

[56] Fidelman S, Zer-Aviv TM, Lange R, Hillard CJ, Akirav I (2018). Chronic treatment with URB597 ameliorates post-stress symptoms in a rat model of PTSD. Eur Neuropsychopharmacol.

[57] Neumeister A, Normandin MD, Pietrzak RH, Piomelli D, Zheng MQ, Gujarro-Anton A, et al. Elevated brain cannabinoid CB1 receptor availability in post-traumatic stress disorder: a positron emission tomography study. Mol Psychiatry. 2013;18:1034–40. 10.1038/mp.2013.61 PMC3752332 23670490

[58] Bedse G, Hartley ND, Neale E, Gaulden AD, Patrick TA, Kingsley PJ, et al. Functional redundancy between canonical endocannabinoid signaling systems in the modulation of anxiety. Bio psychiatry. 2017;82:488–99. 10.1016/j.biopsych.2017.03.002 PMC5585044 28438413

[59] Blankman JL, Simon GM, Cravatt BF. A comprehensive profile of brain enzymes that hydrolyze the endocannabinoid 2-arachidonoylglycerol. Chem Biol. 2007;14:1347–56. 10.1016/j.chembiol.2007.11.006 PMC2692834 18096503

[60] Patel S, Hill MN, Cheer JF, Wotjak CT, Holmes A (2017). The endocannabinoid system as a target for novel anxiolytic drugs. Neurosci Biobehav Rev.

[61] Davidson RJ, Putnam KM, Larson CL (2000). Dysfunction in the neural circuitry of emotion regulation–a possible prelude to violence. Science.

[62] New AS, Buchsbaum MS, Hazlett EA, Goodman M, Koenigsberg HW, Lo J (2004). Fluoxetine increases relative metabolic rate in prefrontal cortex in impulsive aggression. Psychopharmacology.

[63] Bambico FR, Cassano T, Dominguez-Lopez S, Katz N, Walker CD, Piomelli D (2010). Genetic deletion of fatty acid amide hydrolase alters emotional behavior and serotonergic transmission in the dorsal raphe, prefrontal cortex, and hippocampus. Neuropsychopharmacology.

[64] McLaughlin RJ, Hill MN, Bambico FR, Stuhr KL, Gobbi G, Hillard CJ (2012). Prefrontal cortical anandamide signaling coordinates coping responses to stress through a serotonergic pathway. Eur Neuropsychopharm.

[65] Berton O, Nestler EJ (2006). New approaches to antidepressant drug discovery: beyond monoamines. Nat Rev Neurosci.

[66] Bambico FR, Hattan PR, Garant JP, Gobbi G (2012). Effect of delta-9-tetrahydrocannabinol on behavioral despair and on pre- and postsynaptic serotonergic transmission. Prog Neuro-Psychopharmacol Biol Psychiatry.

[67] Franklin JM, Carrasco GA (2012). Cannabinoid-induced enhanced interaction and protein levels of serotonin 5-HT(2A) and dopamine D(2) receptors in rat prefrontal cortex. J Psychopharmacol.

[68] Kolla NJ, Mishra A (2018). The endocannabinoid system, aggression, and the violence of synthetic cannabinoid use, borderline personality disorder, antisocial personality disorder, and other psychiatric disorders. Front Behav Neurosci.

[69] Ferraz L, Portella MJ, Vallez M, Gutierrez F, Martin-Blanco A, Martin-Santos R (2013). Hostility and childhood sexual abuse as predictors of suicidal behaviour in borderline personality disorder. Psychiatry Res.

[70] McGirr A, Paris J, Lesage A, Renaud J, Turecki G (2007). Risk factors for suicide completion in borderline personality disorder: a case-control study of cluster B comorbidity and impulsive aggression. J Clin psychiatry.

[71] Salzman C, Van der Kolk BA, Shader RI (1976). Marijuana and hostility in a small-group setting. Am J Psychiatry.

[72] Bartz JA, Hollander E (2006). The neuroscience of affiliation: forging links between basic and clinical research on neuropeptides and social behavior. Horm Behav.

[73] Striepens N, Scheele D, Kendrick KM, Becker B, Schafer L, Schwalba K (2012). Oxytocin facilitates protective responses to aversive social stimuli in males. Proc Natl Acad Sci USA.

[74] Lischke A, Herpertz SC, Berger C, Domes G, Gamer M (2017). Divergent effects of oxytocin on (para-)limbic reactivity to emotional and neutral scenes in females with and without borderline personality disorder. Soc Cogn Affect Neurosci.

[75] Wei D, Lee D, Cox CD, Karsten CA, Peñagarikano O, Geschwind DH (2015). Endocannabinoid signaling mediates oxytocin-driven social reward. Proc Natl Acad Sci USA.

[76] Schulz SC, Zanarini MC, Bateman A, Bohus M, Detke HC, Trzaskoma Q (2008). Olanzapine for the treatment of borderline personality disorder: variable dose 12-week randomised double-blind placebo-controlled study. Brit J Psychiat.

[77] Uher R, Perlis RH, Placentino A, Dernovšek MZ, Henigsberg N, Mors O (2012). Self-report and clinician-rated measures of depression severity: can one replace the other?. Depression Anxiety.

