# Supplementary material for: Elevated fatty acid amide hydrolase in the prefrontal cortex of borderline personality disorder: a [11C]CURB positron emission tomography study
Source: Neuropsychopharmacology. 2020 Jun 10;45(11):1834–41. doi: 10.1038/s41386-020-0731-y (PMC7608329; doi:10.1038/s41386-020-0731-y)
Supplement: Supplementary file 1 — Supplementary Material [file 41386_2020_731_MOESM1_ESM.docx]

**Supplementary Table 1**

**Comorbid Diagnoses in Borderline Personality Disorder (BPD)** BPD Controls

*n* = 20 *n* = 20

________________________________________________________________________________________

Lifetime major depressive disorder (%) 90 /

Lifetime dysthymic disorder (%) 0 /

Current panic disorder (%) 10 /

Current agoraphobia (%) 10 /

Current specific phobia (%) 5 /

Current social phobia (%) 25 /

Current generalized anxiety disorder (%) 45 /

Current obsessive compulsive disorder (%) 0 /

Current posttraumatic stress disorder (%) 20 /

Previous alcohol use disorder (%) 15 /

Previous cannabis use disorder (%) 0 /

Previous opioid use disorder (%) 0 /

Previous sedative/hypnotic use disorder (%) 0 /

Previous stimulant use disorder (%) 5 /

Previous hallucinogen use disorder (%) 0 /

Previous polysubstance dependence (%) 0 /

Current somatization disorder (%) 0 /

Current pain disorder (%) 0 /

Current undifferentiated somatoform disorder (%) 0 /

Current hypochondriasis (%) 0 /

Current body dysmorphic disorder (%) 0 /

Current anorexia nervosa (%) 0 /

Current bulimia nervosa (%) 0 /

Current eating disorder not otherwise specified (%) 0 /

Current paranoid personality disorder (%) 10 /

Current schizoid personality disorder (%) 0 /

Current schizotypal personality disorder (%) 0 /

Current antisocial personality disorder (%) 10 /

Current histrionic personality disorder (%) 0 /

Current narcissistic personality disorder (%) 0 /

Current avoidant personality disorder (%) 30 /

Current obsessive compulsive personality disorder (%) 10 /

Current dependent personality disorder (%) 15 /

____________________________________________________________________________________________
